# Nanoscale growth of a Sn-guided SiGeSn alloy on Si (111) substrates by molecular beam epitaxy†

**DOI:** 10.1039/d0na00680g

**Published:** 2020-11-19

**Authors:** Liming Wang, Yichi Zhang, Hao Sun, Jie You, Yuanhao Miao, Zuoru Dong, Tao Liu, Zuimin Jiang, Huiyong Hu

**Affiliations:** Wide Bandgap Semiconductor Technology Disciplines State Key Laboratory, School of Microelectronics, Xidian University Xi'an 710071 China lmwang@xidian.edu.cn; School of Physics and Optoelectronic Engineering, Xidian University Xi'an 710071 China hsun@xidian.edu.cn; State Key Laboratory of Surface Physics, Department of Physics, Fudan University Shanghai 200433 China

## Abstract

Here, SiGeSn nanostructures were grown *via* molecular beam epitaxy on a Si (111) substrate with the assistance of Sn droplets. Owing to the thermal effect and the compressive strain induced by a lattice mismatch, Si and Sn atoms were successfully incorporated into the Ge matrix during the Sn-guided Ge deposition process. A low growth temperature of 350 °C produced a variety of SiGeSn nanostructures of different sizes, attributed to the variation of the initial Sn droplet size. Using energy-dispersive X-ray spectroscopy, the Sn, Si and Ge contents of a defect-free SiGeSn nanoisland were approximately determined to be 0.05, 0.09 and 0.86, respectively. Furthermore, as the growth temperature increased past 600 °C, the growth direction of the nanostructure was changed thermally from out-of-plane to in-plane. Meanwhile, the stacked SiGeSn nanowires grown along the 〈112〉 direction remained defect-free, though some threading dislocations were observed in the smooth SiGeSn nanowires along the 〈110〉 direction. These results offer a novel method to grow Si-based SiGeSn nanostructures while possessing important implications for fabricating further optoelectronic devices.

## Abstract 2

Lateral and vertical growth modes of defect-free SiGeSn nanostructures are observed and systematically investigated both experimentally and theoretically.

## Graphical abstract

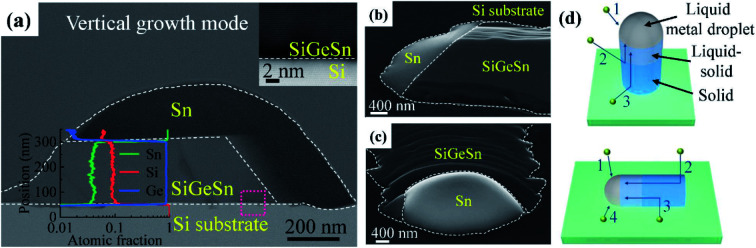

## Introduction

Silicon-based photonics has attracted great interest because of its potential to achieve the monolithic integration of photonic devices with state-of-the-art Si electronic circuits, thereby enabling an oncoming Si-based optoelectronic revolution.^1–6^ While the development of various components for integrated Si photonics is advancing rapidly, there remain many important disadvantages of Si-based photonic devices. For instance, Si is an indirect bandgap semiconductor, and thus it is virtually impossible to realize a high-efficiency Si light source.^7,8^ Moreover, because of the relatively large Si bandgap of 1.12 eV, the responsivity of Si-based photodetectors is extremely low for near-infrared wavelengths,^9,10^ which is a significant obstacle to its application in the infrared optical communication field. In recent years, Ge has been identified as a promising material to achieve high-efficiency group IV infrared photodetectors^11–18^ and light sources,^19–27^ owing to its small bandgap (0.67 eV) and small energy difference (0.13 eV) between the Γ and L valleys (Δ*E*_Γ–L_). Even with all these advantages, the application of Ge to optoelectronics is still restricted owing to its indirect bandgap and the concomitant low efficiency in optoelectronic applications.

Many results have shown that adding Sn atoms into a Ge matrix can transform the material from an indirect semiconductor to a direct one at a Sn concentration of approximately 5–8 at%. By varying the Sn concentration, the bandgap of GeSn alloys can be adjusted depending on the requirements.^28–30^ However, because of the large lattice mismatch between Ge and Sn of around 14 at%, the growth of GeSn alloys with a large percentage of Sn is exceedingly difficult to achieve on Si and Ge substrates. Notably, after the exciting result of an optically pumped GeSn laser reported in 2015,^31^ an electrically pumped GeSn laser has not yet been reported. The most important reason for this delay is precisely because of the poor GeSn crystal quality on Si or Ge substrates. Fortunately, a SiGeSn ternary alloy allows the decoupling of the bandgap and lattice constant and is, therefore, a particularly interesting candidate for Si-based optoelectronic applications. SiGeSn alloys have been successfully grown by chemical vapor deposition (CVD) and molecular beam epitaxy (MBE),^32–37^ and several groups have reported SiGeSn light-emitting diodes, modulators and a multitude of devices for many applications.^38–41^ However, growing SiGeSn on a Si or Ge substrate also suffers from a relatively high dislocation density at the interface, owing to the fact that it is hard to precisely control the content of each element in the growth process. Although it has been reported that growing a thicker SiGeSn layer could prevent dislocations from propagating along the growth direction as the dislocation loop is being formed, the mechanism regarding the formation of dislocations and better growth methods of SiGeSn remain unclear and may limit the development of the SiGeSn technique.^42^

In this paper, we report a novel growth method of Sn-guided SiGeSn nanostructures on Si (111) substrates by MBE. The influences of growth temperature on the growth orientation and the geometric morphology of the nanostructures are systematically investigated. At a low deposition temperature of 350 °C, the lack of uniformity in the Sn droplet size leads to the growth of three types of SiGeSn nanoislands. More interestingly, with increasing growth temperature, lateral SiGeSn nanowires are observed instead of nanoislands, indicating that the SiGeSn nanostructure growth prefers an in-plane growth mode at higher growth temperatures. The nanowires grown along the 〈110〉 direction exhibit flat top surfaces and the Si, Ge, and Sn atoms are distributed evenly along the axial direction, though a considerable amount of threading dislocations is observed in the SiGeSn matrix. In contrast, the nanowires grown along the 〈112〉 direction exhibit a stacked layer structure and are shown to possess good crystallinity with no threading dislocations, which is beneficial for the fabrication of high-performance SiGeSn devices. This proposed method provides an easy technique for controllable growth of SiGeSn nanostructures and a promising possibility for Si-based optoelectronics.

## Experimental

The SiGeSn nanostructures were grown on Si (111) substrates with a solid source MBE system at a base pressure of 2 × 10^−10^ torr. The Si substrates were first cleaned *via* the RCA method and then loaded into an ultra-high-vacuum chamber. After *in situ* thermal desorption of the surface oxide at 980 °C for 20 min, a 50 nm-thick Si buffer layer was deposited at 450 °C and a rate of 0.50 Å s^−1^. Then, the Sn film was deposited on the substrate at 185 °C with a deposition rate of ∼0.067 Å s^−1^. To form Sn droplets, *in situ* annealing was performed for 10 min at various temperatures of 350, 400 or 600 °C. Finally, the SiGeSn nanostructures were grown with high-purity (99.9999 at%) Ge at a deposition rate of 0.1 Å s^−1^. The growth parameters of all the samples are shown in Table 1.

**Table 1:** Summary of the growth parameters for all Sn-guided SiGeSn nanostructure samples

Sample	Substrate	Buffer	Sn deposition thickness (nm)	Annealing temperature (°C)	Deposition material	Deposition thickness (nm)	Ge deposition temperature (°C)
A	Si (111)	Si	10	350	Ge	20	350
A_S1_	Si (100)	Si	10	350	Ge	20	350
A_S2_	Si (100)	Si	10	350	Ge	40	350
A_S3_	Si (111)	Si	10	400	Si	20	400
A_S4_	Si (111)	Si/Ge VS^a^	10	400	Ge	20	400
B	Si (111)	Si	10	400	Ge	20	400
C	Si (111)	Si	4	600	Ge	20	600

a VS represents the virtual substrate.

The surface morphology was characterized using atomic force microscopy (AFM) in tapping mode, scanning electron microscopy (SEM) and high-resolution scanning transmission electron microscopy (STEM). Energy-dispersive X-ray spectroscopy (EDS) in TEM and Raman spectroscopy were employed to study the chemical composition.

## Results and discussion

**Fig. 1:**
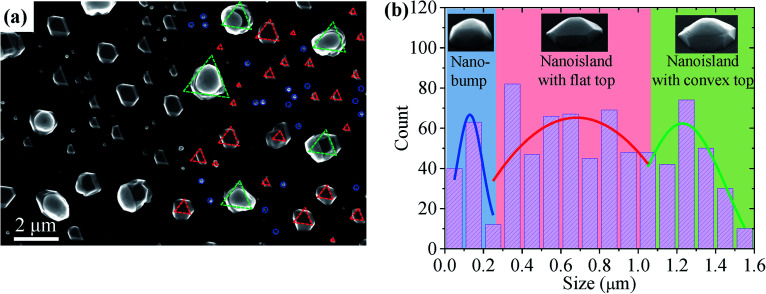
(a) Top-view SEM image of Sn-guided SiGeSn nanostructures grown at 350 °C (sample A). (b) Size distributions of the SiGeSn nanostructures in the SEM images in (a) and Fig. S1a.† (Insets) tilted SEM images of three typical SiGeSn nanostructures.

Fig. 1 shows the top-view SEM image of sample A (Ge deposition on Si (111) with Sn droplets). Three different SiGeSn nanostructures were obtained with the assistance of Sn droplets, as shown in Fig. 1a: (i) rounded SiGeSn nanobumps (marked with blue circles, diameter < 0.2 μm); (ii) SiGeSn islands with a flat top surface (marked with red triangles, the edges of the triangles represent the 〈110〉 direction, diameter in the range of 0.2–1 μm); (iii) SiGeSn islands with a convex top surface (marked with green triangles, the edges of the triangles represent the 〈110〉 direction, diameter > 1 μm). Fig. 1b shows the size distributions of the three types of nanostructures. More morphological details for sample A can be found in Fig. S1 in the ESI.† Two reference samples, including sample A_S1_ (20 nm Ge deposition on Si (100) with Sn droplets) and sample A_S2_ (40 nm Ge deposition on Si (100) with Sn droplets), were grown under the same conditions as sample A to understand the effect of substrate orientation. Fig. S2a and b (see the ESI)† show that islands were obtained on the entire substrate surface of samples A_S1_ and A_S2_, respectively, where the growth mode of the islands for both reference samples was Stranski–Krastanov growth. Additionally, to clarify the formation mechanism of the nanostructures found on sample A, another two reference samples, including sample A_S3_ (Si deposition on Si (111) with Sn droplets) and sample A_S4_ (Ge virtual substrate is introduced between the Sn droplet and Si substrate), were grown. No nanostructures such as those on sample A were observed on sample A_S3_ except for Sn droplets on the surface, as shown in Fig. S3 (see the ESI).† The Si deposition produced a thin film on the Si (111) surface owing to the perfect lattice match. Furthermore, the SEM images of sample A_S4_ given in Fig. S4 (see the ESI)† show that a morphology such as that of sample A is observed, including the flat top surface, though sample A_S4_ with a Ge virtual substrate exhibits a rough surface and a poor crystal quality. According to these results, the growth of Sn-guided nanostructures in this work is considered to be related to the compressive strain. When growing Si on a Si (111) substrate or growing Ge on the Ge virtual substrate, the strain between the deposited atoms and the substrate is diminished or becomes zero, thereby resulting in an unexpected result. For sample A, however, the compressive strain from the lattice mismatch between Ge and Si (111) results in the formation of several types of nanostructures with the assistance of Sn. In this process, it was possible to melt the Si and Sn atoms into the Ge matrix to obtain the SiGeSn alloy that was expected.

**Fig. 2:**
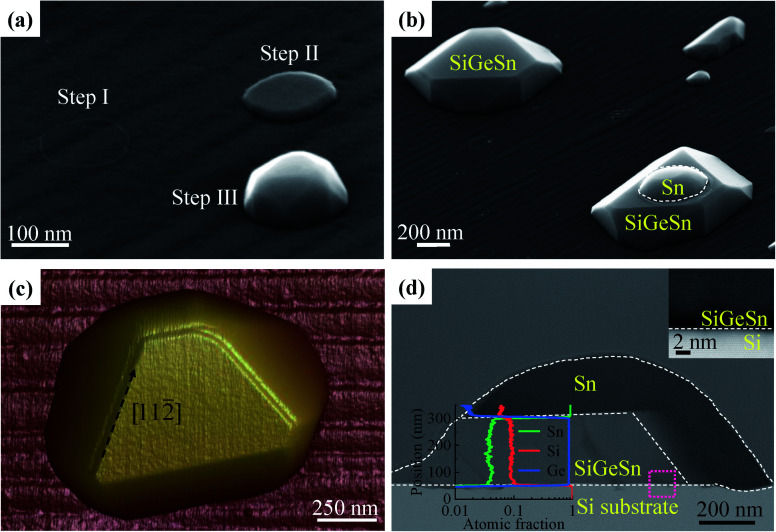
(a) Tilted SEM image illustrating the growth process of SiGeSn nanobumps with a diameter of ∼100 nm. (b) Tilted SEM image of SiGeSn nanoislands with a flat top surface (upper left) and convex top surface (lower right). (c) AFM image of the SiGeSn nanoisland with a flat top surface. (d) Cross-sectional bright-field STEM image of the SiGeSn nanoisland with a convex top surface. (Plot inset) EDS line scan from the Si substrate to the Sn droplet through the centre of the nanoisland and (image inset) high-resolution bright-field STEM image of the SiGeSn nanoisland with a convex top surface at the SiGeSn/Si substrate interface (acquired at the pink rectangle area).

Furthermore, we ascribe the formation mechanism of the three different nanostructures in sample A, including nanoislands and nanobumps, to the different sizes of the initial Sn droplets. For the small-diameter Sn droplet (<0.2 μm), the Sn-guided SiGeSn growth process stops quickly because of the consumption or shedding of the small Sn droplets, and thus small SiGeSn nanobumps are obtained. Fig. 2a shows the entire evolution process of a ∼100 nm diameter SiGeSn nanobump. At the starting point of growth, Ge is decomposed and dissolved in the Sn droplet leading to Ge supersaturation and subsequent nucleation/film formation underneath the Sn droplet *via* layer-by-layer growth, which represents typical vapor–liquid–solid (VLS) growth.^43,44^ During the growth process the Sn atoms are consumed, which leads to a decreasing metal droplet diameter, whereupon the small nanobump formation stops when the Sn droplet is drained. For the large-diameter Sn droplets, a large number of Sn atoms are present in the Sn droplet and thus the consumption of Sn does not significantly affect the Sn droplet diameter. Thus, larger SiGeSn islands with diameters greater than 0.2 μm can ultimately be formed with the assistance of the large Sn droplets. As shown in Fig. 2b, two types of SiGeSn islands of the same size can be observed, including islands with a flat top surface and islands with a convex top surface. After the Sn droplet is shed from the top surface, the SiGeSn islands exhibit a flat top surface, where many of the sidewalls of the island exhibit the different lattice planes of a perfect crystal. Similar nanostructures were obtained in III–V nanowire growth^45,46^ and the small wetting angle is related to the consumption of Sn atoms in the Sn droplet. During the growth process, Sn atoms in the Sn droplet were consumed which leads to the reduction of the volume of the Sn droplet, and as a result the wetting angle is reduced. If the wetting angle is smaller than a certain angle, the growth of SiGeSn nanoislands stops earlier. In consequence, a Sn droplet with a small wetting angle could be found on top of the SiGeSn nanoisland. Moreover, Fig. 2b shows the tilted SEM image of sample A, and hence the wetting angle looks smaller than it is. These planar facets can be observed in the AFM image of a flat-top nanoisland shown in Fig. 2c, which is in good agreement with other reports of Ge islands on Si (111). Furthermore, Fig. 2d shows a cross-sectional bright-field STEM image of a SiGeSn nanoisland with a convex top surface. No threading dislocations are observed in the SiGeSn island, indicating the good crystal quality of the island. An EDS line scan from the substrate to the top of a nanoisland confirms that the contents of Sn, Si and Ge in the defect-free SiGeSn island are 0.05, 0.09 and 0.86, respectively. Additionally, the full shedding process of a Sn droplet from a huge SiGeSn island is displayed in Fig. S5 in the ESI.† This shedding process reveals the weak contact between the Sn droplet and SiGeSn island.

**Fig. 3:**
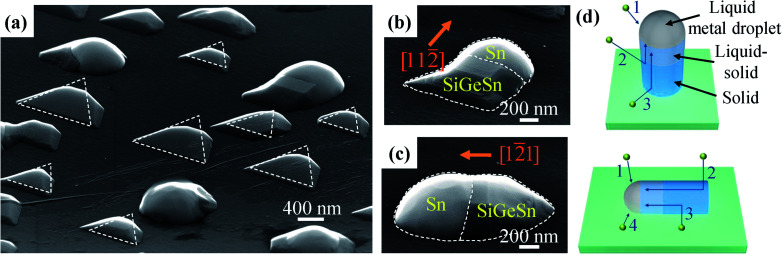
(a) Tilted SEM image of Sn-guided SiGeSn nanostructures grown at 400 °C. (b and c) SEM images of a lateral SiGeSn nanostructure grown along the (b) [112̄] and (c) [12̄1] directions. (d) Schematic diagram showing nanostructure growth by the VLS method. The growth of the SiGeSn nanostructure is driven by 4 mechanisms: (1) adsorption at the droplet surface; (2) the direct impingement of atoms onto the sidewalls with subsequent diffusion to the droplet; (3) diffusion of surface adatoms to the NW sidewalls and then to the droplet; (4) direct diffusion of surface adatoms to the droplet.

Fig. 3 presents the SEM images of sample B, grown by Ge deposition on Si (111) with Sn droplets at 400 °C. As the Sn annealing temperature and Ge growth temperature were increased from 350 to 400 °C, the size of the SiGeSn nanoislands became more uniform. This result can be explained with the formation of Sn droplets of increased uniformity. In the annealing process, the Sn film existing on the Si (111) was broken apart and the Sn atoms were reassembled into Sn droplets. The larger amount of energy present at higher annealing temperatures led to a longer average migration distance for the Sn atoms, and consequently Sn droplets with increased uniformity were obtained. Interestingly, the SiGeSn islands exhibited a lateral growth trend, as shown by the two islands grown along the 〈112〉 direction in Fig. 3b and c.

This temperature-dependent growth of a nanowire signifies that the growth temperature can modify the metal–semiconductor interface and thus alter the growth direction of the nanowire. During the growth process, the nanostructure can be understood as a column containing a solid nanowire, a liquid–solid (LS) melt region, and a liquid metal droplet, as depicted in Fig. 3d. The kinetics of the VLS growth of the out-of-plane and in-plane grown SiGeSn nanostructure are also illustrated in Fig. 3d. Within the LS region, atoms are mobile and may be rearranged to modify the nanowire growth direction to minimize energy. At a higher growth temperature, the LS region is sufficiently thick that the nanostructure can grow along the direction of minimum energy, such as the lateral nanowire from sample B. In contrast, at a low growth temperature, the LS region is insufficiently thick for a direction change. Thus the nanowire growth remains in the out-of-plane 〈111〉 direction even though this is a growth direction of higher energy, as exemplified by the nanoislands in sample A. Besides this effect, the growth temperature can also modify the surface energy, and thus change the geometry of the Sn droplet which leads to the change in the growth direction of the nanowire.

**Fig. 4:**
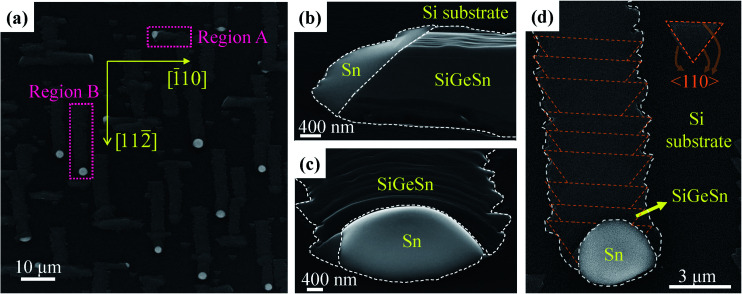
(a) Top-view SEM image of Sn-guided SiGeSn nanowires grown at 600 °C. Tilted SEM images of the nanowire grown along (b) [1̄10] and (c) [112̄] directions, respectively. (d) Top-view SEM image of the Sn-guided SiGeSn nanowire grown along the [112̄] direction.

To achieve an actual SiGeSn nanowire, we optimized the growth process of Sn-guided SiGeSn and obtained SiGeSn nanowires aligned horizontal to the substrate. The growth conditions are given for sample C in Table 1. Fig. 4 shows the morphology of the SiGeSn nanowire, where the bidirectional growth can be clearly seen in the SEM images. More morphological details for sample C can be found in Fig. S6 and S7 in the ESI.† Interestingly, a noticeable difference in growth rates between the two directions (〈110〉 and 〈112〉 directions) can be found. The growth rate along 〈110〉 is less than that along 〈112〉, resulting in short nanowires grown along 〈110〉 (average length ∼ 9.4 μm, as shown in Fig. S8a in the ESI†) and long nanowires grown along 〈112〉 (average length ∼ 18.8 μm, as shown in Fig. S8b in the ESI†). This observation of Sn-guided SiGeSn nanowires grown along the 〈110〉 direction is absent in previous studies, which show only nanowires grown along the 〈112〉 direction. At a low growth temperature, as shown in Fig. 3 for sample B, lateral nanowires are grown along the 〈112〉 direction because of the minimum energy in this direction. However, at a high growth temperature of 600 °C for sample C, the atoms possess a higher energy than that at a low growth temperature, permitting the nanowires to grow along the preferred 〈112〉 direction (*i.e.*, minimum energy direction) and also along the 〈110〉 direction (*i.e.*, second-minimum energy direction).

The lateral growth mode of the Sn-guided nanowire can be explained by the thermal effect on the metal/semiconductor interface. As mentioned above, whether the growth direction of the nanowire will change largely depends on the structure at the LS interface between the Sn droplet and the nanowire, as shown in Fig. 3d. At a high growth temperature of 600 °C, the LS region is sufficiently thick and thus the atoms in this region can be rearranged to modify the nanowire growth direction to 〈110〉 or 〈112〉, which represent the minimum energy growth directions. In other words, the growth direction of the nanostructure can be thermally changed with increasing temperature from out-of-plane (*i.e.*, lower growth temperature, such as 350 °C for sample A) to in-plane (*i.e.*, higher growth temperature, such as 600 °C for sample C). This mechanism was also validated using Si nanowires grown on a Si (111) substrate at temperatures between 450 and 600 °C, where the yields of vertical nanowires decreased with increasing temperature.^47^

The nanowires that propagate along the 〈110〉 direction (Fig. 4b) exhibit a flat top surface, while the nanowires that propagate along the 〈112〉 direction exhibit a stacked layer structure (Fig. 4c). The nanowire grown along 〈112〉 comprises multiple triangular nanoislands, as marked by orange dotted lines in Fig. 4d, which is commonly seen in Ge growth on Si (111).^48,49^ In this stacked layer structure growth, the first triangular nanoisland grows next to the Sn droplet owing to the low nucleation energy, whereupon the next newly formed triangular nanoisland grows between the Sn droplet and the first triangular nanoisland, which causes the Sn droplet to move along an opposite direction. Finally, the all triangular nanoislands overlap to form a toothed nanowire.

**Fig. 5:**
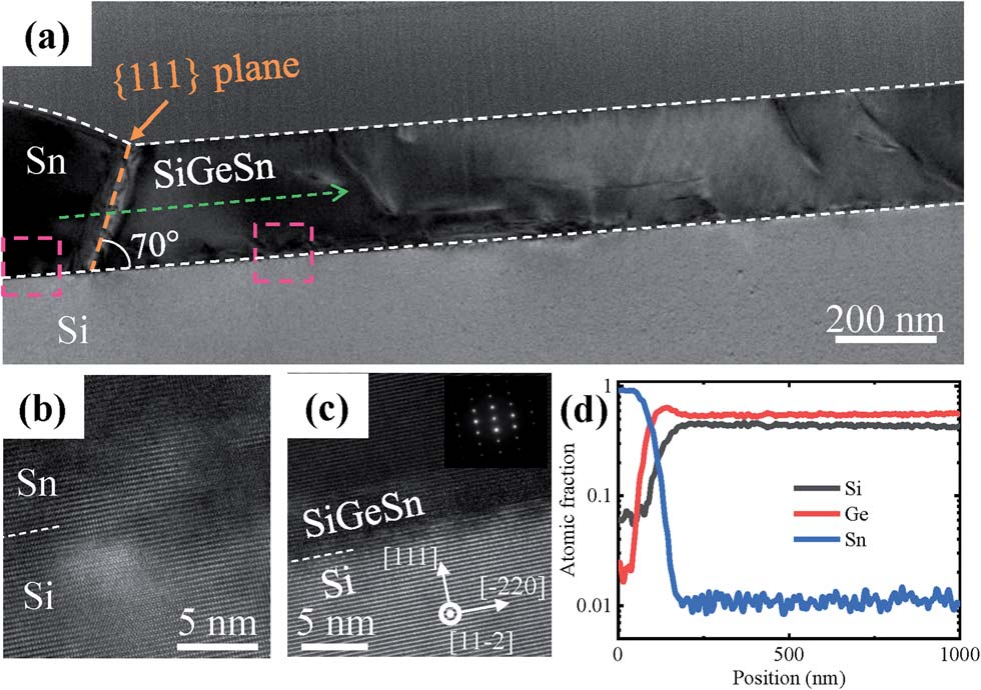
(a) Cross-sectional bright-field STEM image of the SiGeSn nanowire grown along the 〈110〉 direction, and the orange dashed line is parallel to the {111} planes of the SiGeSn nanowire. High-resolution bright-field STEM images of (b) Sn/Si interface and (c) SiGeSn/Si interface, respectively, marked by left and right pink rectangles in (a). (Inset) the corresponding SAED pattern. (d) EDS line scan along the green dotted arrow in (a).

Fig. 5 shows the bright-field STEM and EDS line scan results of the nanowire grown along the 〈110〉 direction (region A marked in Fig. 4a). The thickness of the nanowire is approximately 200 nm and a large number of threading dislocations are distributed in the entire SiGeSn nanowire, as shown in Fig. 5a. A 70° angle exists between the two interfaces, illustrated by an orange dashed line in Fig. 5a; this signifies that the supersaturated SiGeSn is separated from the Sn by {111} planes. Fig. 5b and c present the high-resolution bright-field STEM images of the Sn droplet/Si interface and SiGeSn nanowire/Si interface, respectively. A coherent interface is formed between the Si and SiGeSn nanowire along the {111}-Si planes, while no clear interface is found between the Sn droplet and Si substrate. Moreover, as shown in Fig. 5d, the EDS line scan along the SiGeSn nanowire indicates that the Si, Ge and Sn are evenly distributed along the nanowires. The contents of Si and Ge are ∼0.44 and 0.55, respectively. However, the Sn content is low (∼0.01) in the SiGeSn nanowire owing to the high growth temperature of 600 °C. Furthermore, the Raman spectra at different positions in the nanowires grown along the 〈110〉 direction are shown in Fig. S9 in the ESI.† The profiles of the Raman spectra from all positions showed good correspondence, indicating that the contents of Si, Ge and Sn are nearly equivalent in the SiGeSn nanowire grown along the 〈110〉 direction.

**Fig. 6:**
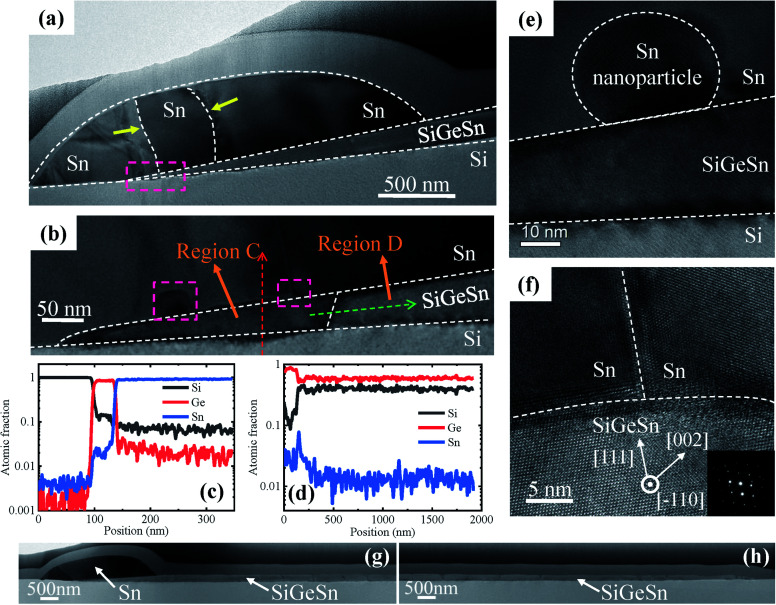
(a) Cross-sectional bright-field STEM image of the SiGeSn nanowire grown along 〈112〉. (b) Enlarged view of the cross-sectional bright-field STEM image at the front of the SiGeSn nanowire grown along 〈112〉 (marked by the pink rectangle in (a)). (c and d) EDS line scan along the (c) red and (d) green dotted arrows in (b). (e and f) High-resolution bright-field STEM images of (e) Sn nanoparticle in the front part of the Sn droplet and (f) the interface within the Sn droplet and between the Sn droplet and SiGeSn nanowire (marked by pink rectangles in (b)). (Inset) the corresponding SAED pattern. Bright-field STEM image of the (g) front and (h) back of the SiGeSn nanowire grown along the 〈112〉 direction.

Fig. 6 shows the cross-sectional bright-field STEM images and EDS line scan results from the nanowire grown along the 〈112〉 direction (region B marked in Fig. 4a). Compared with that grown along 〈110〉, the cross-section of the nanowire grown along 〈112〉 exhibits a wedge shape. We note that no threading dislocations are observed in the SiGeSn nanowire in the cross-sectional bright-field STEM image (Fig. 6a). Two obvious interfaces (marked by yellow arrows in Fig. 6a) within the Sn droplet can be observed, which demonstrates that this large-sized Sn droplet originates from several small Sn droplets merging during the annealing process of the Sn film. Fig. 6c and d respectively show the EDS line scans across the wedge-shaped SiGeSn nanowire (red dotted arrow in Fig. 6b) and along the axial direction (green dotted arrow in Fig. 6b). The front part of the wedge-shaped nanowire exhibits a higher Ge content (∼0.84 in region C) than the rear part (∼0.55 in region D), while the Si content in the front part (∼0.13 in region C) is less than that in the rear part (∼0.44 in region D). Additionally, the Sn content in the SiGeSn nanowire is ∼0.03 and ∼0.01 at the front and rear parts, respectively, and has a peak value of ∼0.08 at the interface between regions C and D. The EDS spectrum of the SiGeSn nanowire grown along the 〈112〉 direction can be found in Fig. S10 in the ESI,† and a Sn peak is observed which confirms the existence of Sn atoms in the SiGeSn nanowire. Furthermore, we note that a Sn nanoparticle is observed in the front part of the Sn droplet near the Sn droplet/SiGeSn nanowire interface as shown in Fig. 6e, which can be explained with the unique nucleation site. Fig. 6f shows an interface existing within the Sn droplet and the interface between the Sn droplet and SiGeSn nanowire. Simultaneously, the image in Fig. 6f confirms a defect-free SiGeSn nanowire with a high crystal quality, which is required for the fabrication of high-performance SiGeSn devices. Fig. 6g and h illustrate the bright-field STEM image of the front and back of the SiGeSn nanowire grown along the 〈112〉 direction, and more details of the nanowire can be found in Fig. S11 in the ESI.† These images indicate that high crystal quality SiGeSn nanowires were successfully synthesized on the Si substrate.

**Fig. 7:**
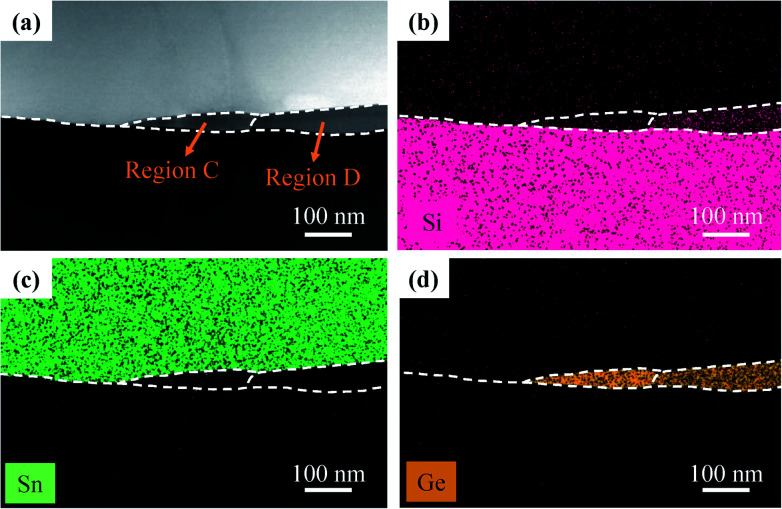
(a) High-angle annular dark field (HAADF) cross-sectional STEM image of the SiGeSn nanowire grown along 〈112〉, and (b–d) corresponding EDS maps of (b) Si, (c) Sn and (d) Ge distributions.

Corresponding to the HAADF STEM results, EDS mapping of the SiGeSn nanowire grown along the 〈112〉 direction is shown in Fig. 7. The images reconfirm that Si atoms are primarily distributed in the rear part of the nanowire while Ge atoms are primarily distributed in the front part of the nanowire, as shown in Fig. 7b and d, respectively. Owing to the low Sn content in the SiGeSn nanowire and the low accuracy for element detection, very few Sn atoms can be observed in the SiGeSn nanowire, as shown in Fig. 7c.

## Conclusions

We present a viable VLS approach for the preparation of SiGeSn nanostructures on Si (111) substrates with the assistance of Sn droplets. At a growth temperature of 350 °C, three types of SiGeSn nanostructures were obtained, including SiGeSn nanobumps and SiGeSn nanoislands with flat top surfaces or convex top surfaces. The contents of Sn and Si in these nanostructures reached ∼0.05 and ∼0.09, respectively. Modulation of the SiGeSn nanowire orientation *via* the change of growth temperature was attributed to the thermal effect on the metal/semiconductor interface. Furthermore, lateral growth of Sn-guided SiGeSn nanowires was achieved at a higher growth temperature of 600 °C, where a bidirectional growth morphology was observed. The SiGeSn nanowires along the 〈110〉 direction presented a flat top surface, while SiGeSn nanowires along the 〈112〉 direction exhibited a stacked layer structure. The structural analysis confirmed that the SiGeSn nanowires along 〈112〉 possessed a predominantly defect-free lattice. These results reveal an effective method to synthesize high-quality SiGeSn nanostructures including nanobumps, nanoislands, and nanowires, and can provide useful support for the application of SiGeSn nanostructures in optoelectronic devices.

## Conflicts of interest

There are no conflicts to declare.

## Supplementary Material

NA-003-D0NA00680G-s001
