# Supplementary material for: Nanoscale growth of a Sn-guided SiGeSn alloy on Si (111) substrates by molecular beam epitaxy
Source: Nanoscale Adv. 2020 Nov 19;3(4):997–1004. doi: 10.1039/d0na00680g (PMC9419757; doi:10.1039/d0na00680g)
Supplement: NA-003-D0NA00680G-s001 [file NA-003-D0NA00680G-s001.pdf]

Electronic Supplementary Material (ESI) for Nanoscale Advances.
This journal is © The Royal Society of Chemistry 2020

Nanoscale Growth of Sn-guided SiGeSn Alloy on Si (111) Substrates by Molecular Beam Epitaxy

Liming Wang, Yichi Zhang, Hao Sun, Jie You, Yuanhao Miao, Zuoru Dong, Tao Liu, Zuimin Jiang and Huiyong Hu

Fig. S1 (a) and (b) present the top-view and tilted SEM images of Sample A (Ge deposition on Si (111) with Sn droplets). Three typical structures, the rounded SiGeSn nanobump, the SiGeSn islands with flat top surface and the SiGeSn islands with convex top surface, are observed in this sample.

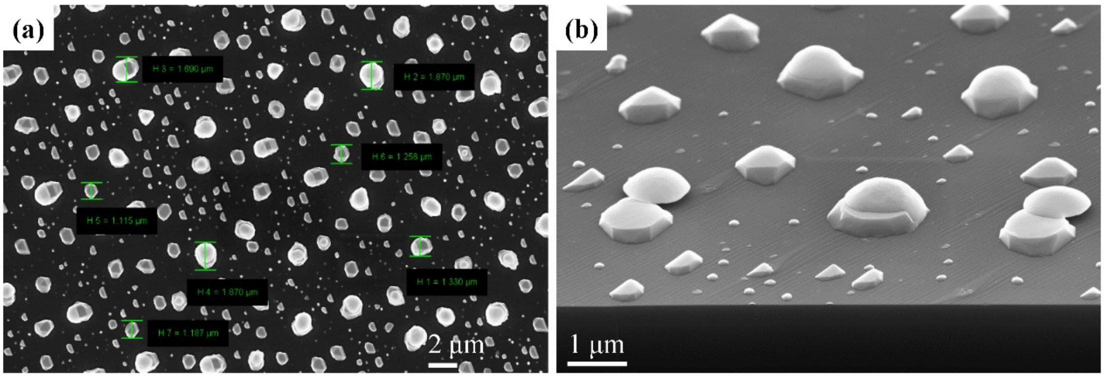

Fig. S1 (a) Top-view and (b) tilted SEM images of SiGeSn nanostructures grown at 350 °C.

Fig. S2 presents the tilted SEM images of (a) Sample A_{S1} (20 nm Ge deposition on Si (100) with Sn droplets) and (b) Sample A_{S2} (40 nm Ge deposition on Si (100) with Sn droplets). Sn droplets were failed to assist the growth of SiGeSn nanostructures on Si (100) substrates.

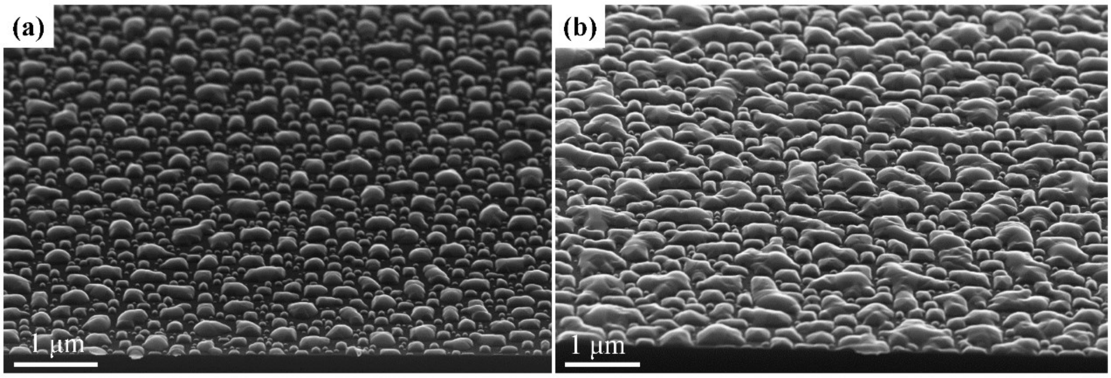

Fig. S2 Tilted SEM images of (a) Sample A_{S1} (20 nm Ge deposition on Si (100) with Sn droplets) and (b) Sample A_{S2} (40 nm Ge deposition on Si (100) with Sn droplets)

Fig. S3 presents (a) top-view and (b) tilted SEM images of Sample A_{S3} (Si deposition on Si (111) with Sn droplets). No Sn-guided growth was observed on the surface of Sample A_{S3}. The high-resolution tilted SEM image, shown in Fig. S3 (c)-(e), confirmed the failure of Sn-guided growth.

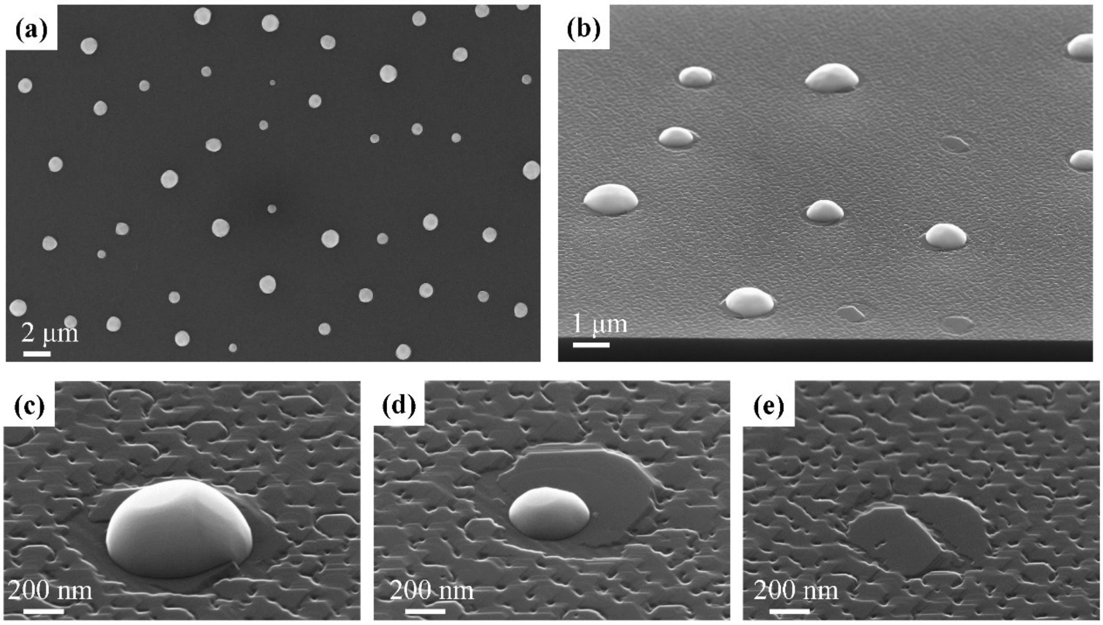

Fig. S3 (a) Top-view and (b) tilted SEM images of Sample A_{S3} (Si deposition on Si (111) with Sn droplets). (c)-(e) High resolution tilted SEM image of Sample A_{S3}.

Fig. S4 shows the SEM images Sample A_{S4} (Ge deposition on Si (111) with Ge VS buffer and Sn droplets). A similar morphology including the flat top surface nanoislands to Sample A is observed, but the surface is rough and the crystal quality looks poor for the Sample A_{S4} with a Ge virtual substrate.

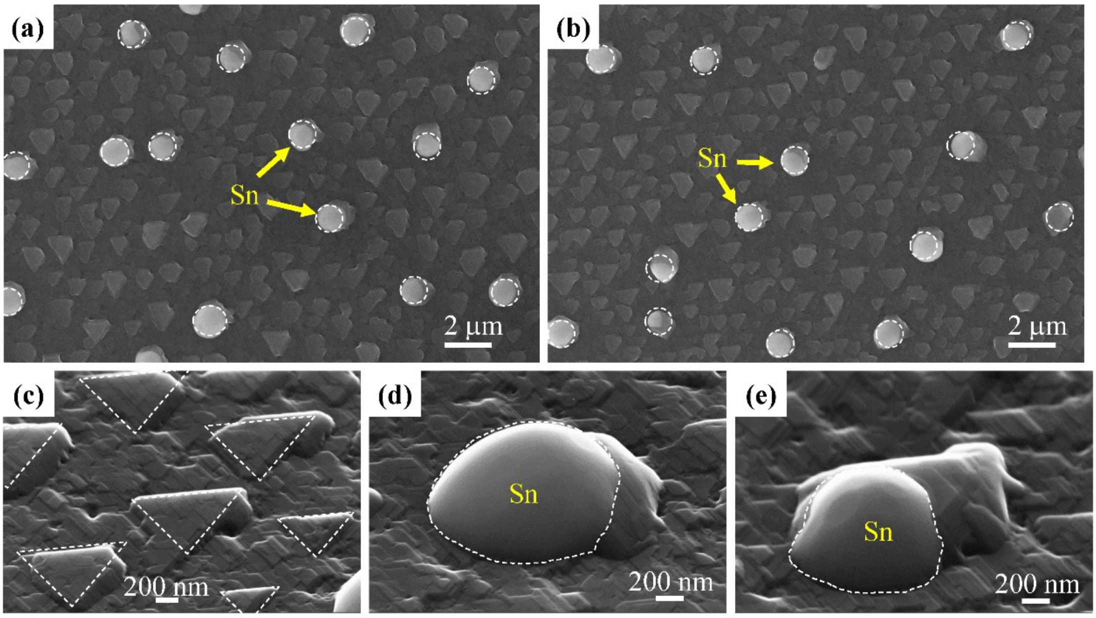

Fig. S4 (a) and (b) Top-view SEM images at different locations of Sample B2. (c) Details of the tilted SEM morphological between two Sn droplets. (d) and (e) Details of the tilted SEM morphological of Sn droplet.

Fig. S5 shows the full shedding process of a Sn droplet from a huge SiGeSn island.

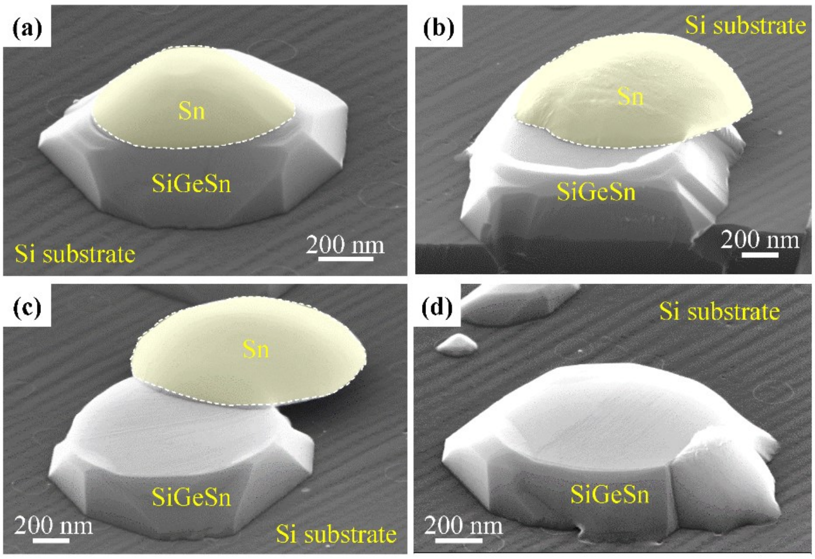

Fig. S5. Full shedding process of Sn droplet from a SiGeSn nanoisland. (a) Before the shedding process starts. (b) Half of the Sn droplet has been shed. (c) More than half of the Sn droplet has been shed. (d) Sn droplet is completely shed from SiGeSn nanoisland.

Fig. S6 shows the tilted SEM image of Sample C and microscope images of of sample C are shown in Fig. S7.

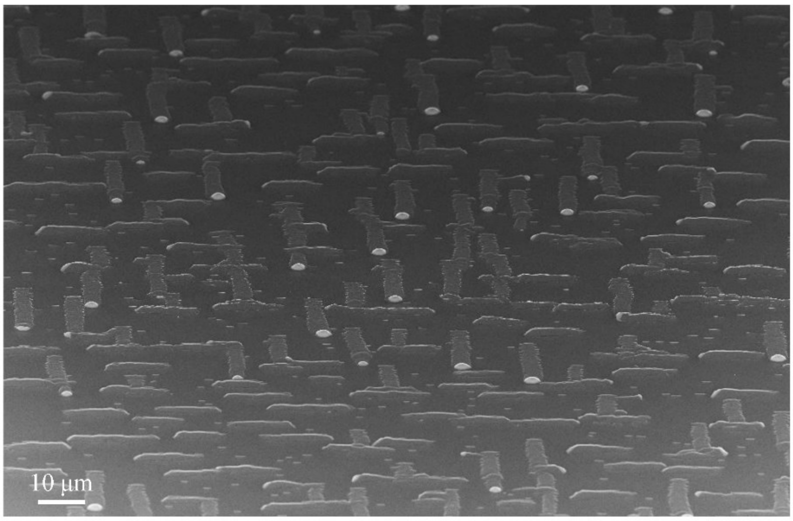

Fig. S6 Tilted SEM image of of Sn-guided SiGeSn nanowires grown at 600 °C (Sample C)

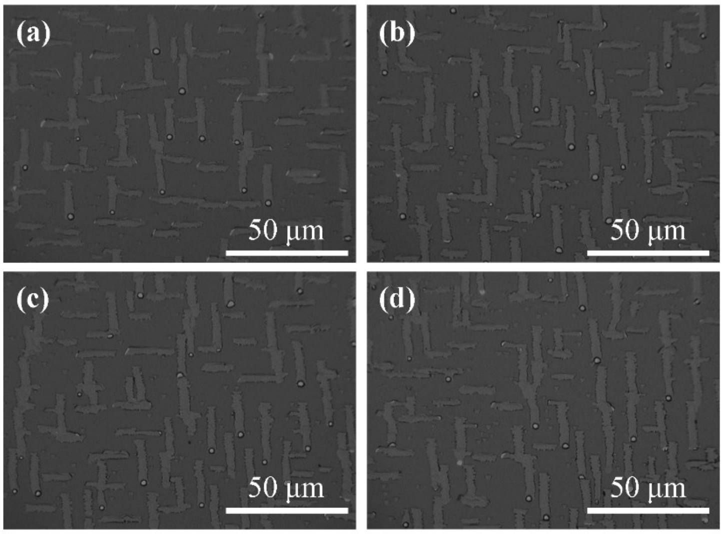

Fig. S7 Microscope images of of Sn-guided SiGeSn nanowires grown at 600 °C (Sample C)

Fig. S8 (a) and (b) show the length distribution of SiGeSn nanowire grown along <110> and <112> directions, respectively. This result demonstrates that the lengths of SiGeSn nanowires grown along <110> and <112> direction are ～9.4 and ～18.8 μm, respectively.

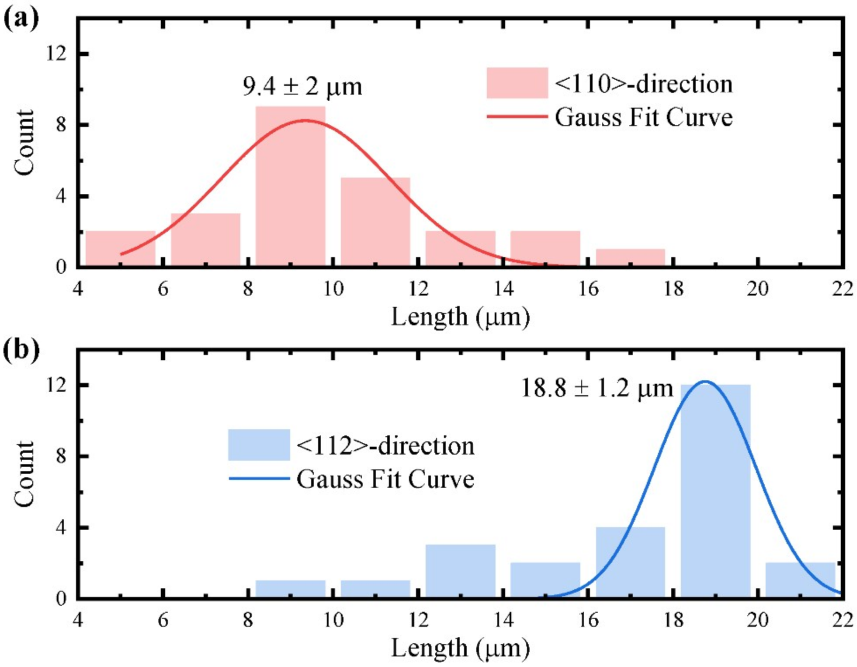

Fig. S8 The length distribution of SiGeSn nanowires grown along (a) <110> and (b) <112> directions.

Fig. S9 shows the Raman spectra at different positions of nanowire grown along <110> direction. This result implies that the contents of Sn, Si and Ge are almost the same in SiGeSn nanowire grown along <110> direction.

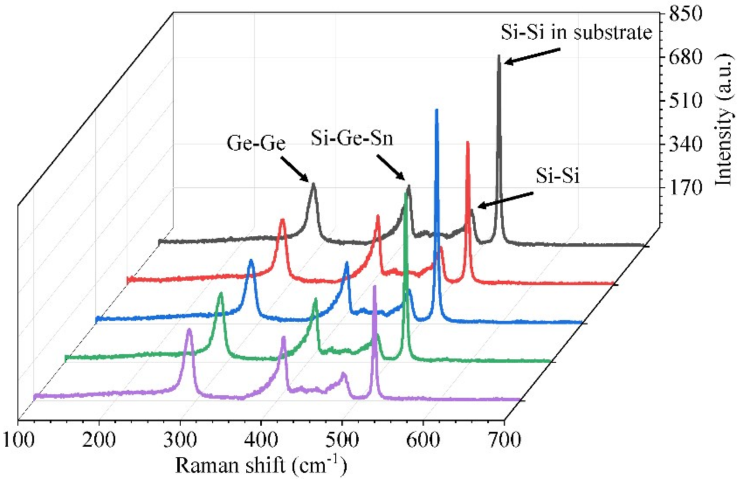

Fig. S9 Raman spectra at different positions of nanowire grown along <110> direction.

EDS spectrum of SiGeSn nanowire is shown in the Fig. S10 below. Si, Ge and Sn peak could be found in the spectra indicating that Si, Ge and Sn were contained in the nanowire. The Cu peaks were from the Cu grid for TEM measurement. This result confirmed the incorporation of Sn into the nanowire.

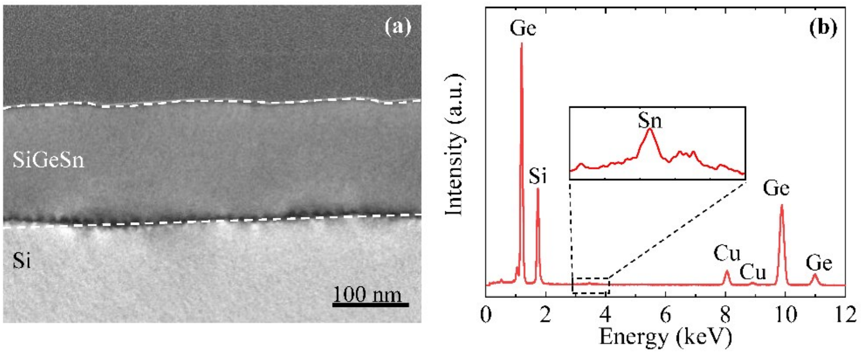

Fig. S10 (a) Cross-sectional bright-field STEM image of the SiGeSn nanowire grown along <112>. (b) The corresponding EDS spectrum.

As shown in the Fig. S11, the nanowire shows high quality and a few dislocations are observed in the nanowire, while a large amount of dislocations are observed in the back of the nanowire. As shown in Fig. S11f, massive dislocation could be observed due to the lattice mismatch between SiGeSn and Si (because the nucleation appears at the interface between Sn droplet and Si substrate at the start point of the nanowire growth). As the nanowire continue to grow, the nucleation of SiGeSn appear at the interface of SiGeSn nanowire and Sn droplet, instead of at the interface of Si sub and Sn droplet, therefore the lattice mismatch is negligible. As a result, the SiGeSn nanowire grown along <112> shows a good crystal quality.

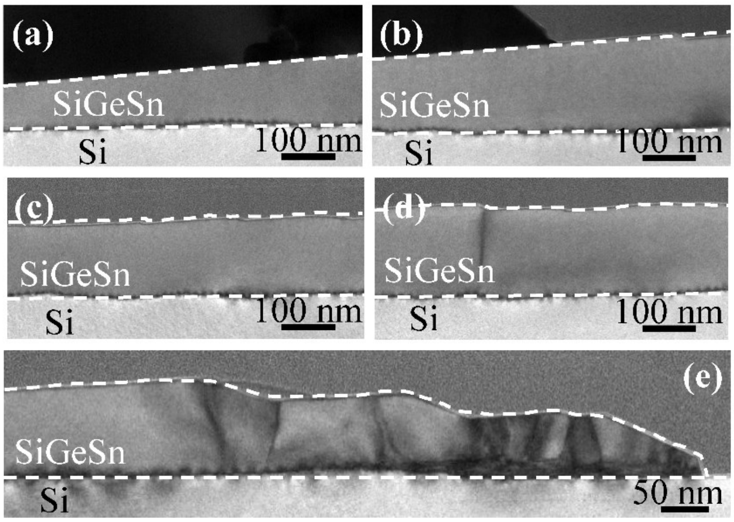

Fig. R11 Bright-field STEM images of SiGeSn nanowire grown along <112> direction: (a) and (b) the front of nanowire; (c) and (d) middle of the nanowire; (e) back of the nanowire.
